# Prognostic significance of reduced handgrip strength in patients with unresectable hepatocellular carcinoma receiving HAIC combined with targeted immunotherapy

**DOI:** 10.3389/fimmu.2025.1672519

**Published:** 2025-12-11

**Authors:** Xin-Ze Wang, Ping Wang, Xing Lv, Zi-Long Zhang, Er-Lei Zhang, Gui-Bao Ji

**Affiliations:** 1 Department of Hand Surgery, Wuhan Fourth Hospital, Wuhan, China; 2 Department of Hepatobiliary-Pancreatic and Hernia Surgery, Wuhan Fourth Hospital, Wuhan, China; 3 Hepatic Surgery Center, Tongji Hospital, Tongji Medical College, Huazhong University of Science and Technology, Wuhan, China; 4 Research Laboratory and Hepatic Surgery Center, Department of Surgery, Tongji Hospital, Tongji Medical College, Huazhong University of Science and Technology, Wuhan, China; 5 Hubei Key Laboratory of Hepato-Pancreato-Biliary Diseases, Wuhan, China

**Keywords:** handgrip strength, sarcopenia, hepatocellular carcinoma, HAIC, targeted immunotherapy, prognosis

## Abstract

**Background:**

Hepatic arterial infusion chemotherapy (HAIC) combined with targeted immunotherapy has emerged as a key therapeutic option for advanced (or unresectable) hepatocellular carcinoma (HCC). Nevertheless, treatment efficacy varies significantly among individuals. Sarcopenia, characterized by loss of muscle mass and strength, may adversely affect therapeutic outcomes and patient prognosis. This study investigates the clinical relevance of sarcopenia in patients undergoing HAIC combined with targeted immunotherapy.

**Methods:**

A total of 265 patients with unresectable HCC who received HAIC combined with targeted immunotherapy were retrospectively enrolled in this study and divided into two groups (sarcopenic and non-sarcopenic group). Sarcopenia was defined based on handgrip strength (HGS), with a cutoff value of less than 28kg(male) and 18kg(female). Overall survival (OS) and progression-free survival (PFS) were compared between groups using the log-rank test and multivariate Cox proportional hazards models. Additionally, propensity score matching (PSM) was applied to minimize baseline differences and enhance comparability. Then, differences in treatment response, survival outcomes, and adverse events between the sarcopenic and non-sarcopenic groups were evaluated using appropriate statistical analyses.

**Results:**

Patients in sarcopenia group exhibited significantly poorer OS and PFS compared to those with non-sarcopenic group. Sarcopenia was also associated with lower objective response rates. Multivariate analysis confirmed that sarcopenia was an independent prognostic factor for poor outcomes.

**Conclusion:**

Sarcopenia is a significant predictor of poor prognosis in patients with unresectable HCC treated with HAIC combined with targeted immunotherapy. Incorporating HGS assessment into clinical practice may help optimize individualized treatment strategies and enhance patient management.

## Introduction

Hepatocellular carcinoma (HCC) ranks as the sixth most commonly diagnosed cancer worldwide and is the third leading cause of cancer-related death (1). Due to its insidious onset, over 70% of HCC cases are diagnosed at an advanced stage, where curative surgical intervention is no longer feasible (2). In recent years, the combination of hepatic arterial infusion chemotherapy (HAIC) with targeted and immune therapies has demonstrated promising outcomes in treating unresectable HCC (3–5). However, owing to significant heterogeneity, there is a pressing need to identify simple and practical prognostic indicators (6). Sarcopenia is a syndrome marked by age-related loss of skeletal muscle mass, strength, or physical function (7), and it has been associated with poor outcomes across multiple cancer types (8–10). As a key indicator of sarcopenia (11), handgrip strength is easy to measure, highly repeatable, and especially suitable for HCC patients with impaired liver function (12). According to the Asian Working Group for Sarcopenia (AWGS) 2019 guidelines, handgrip strength is considered a core diagnostic criterion for sarcopenia (13). This study aims to investigate the effect of sarcopenia, defined by reduced handgrip strength, on the treatment efficacy, survival outcomes, and safety in patients with unresectable HCC undergoing HAIC combined with targeted immunotherapy, providing valuable evidence to support clinical stratification and personalized therapeutic approaches.

## Methods

### Patients

**Figure 1:**
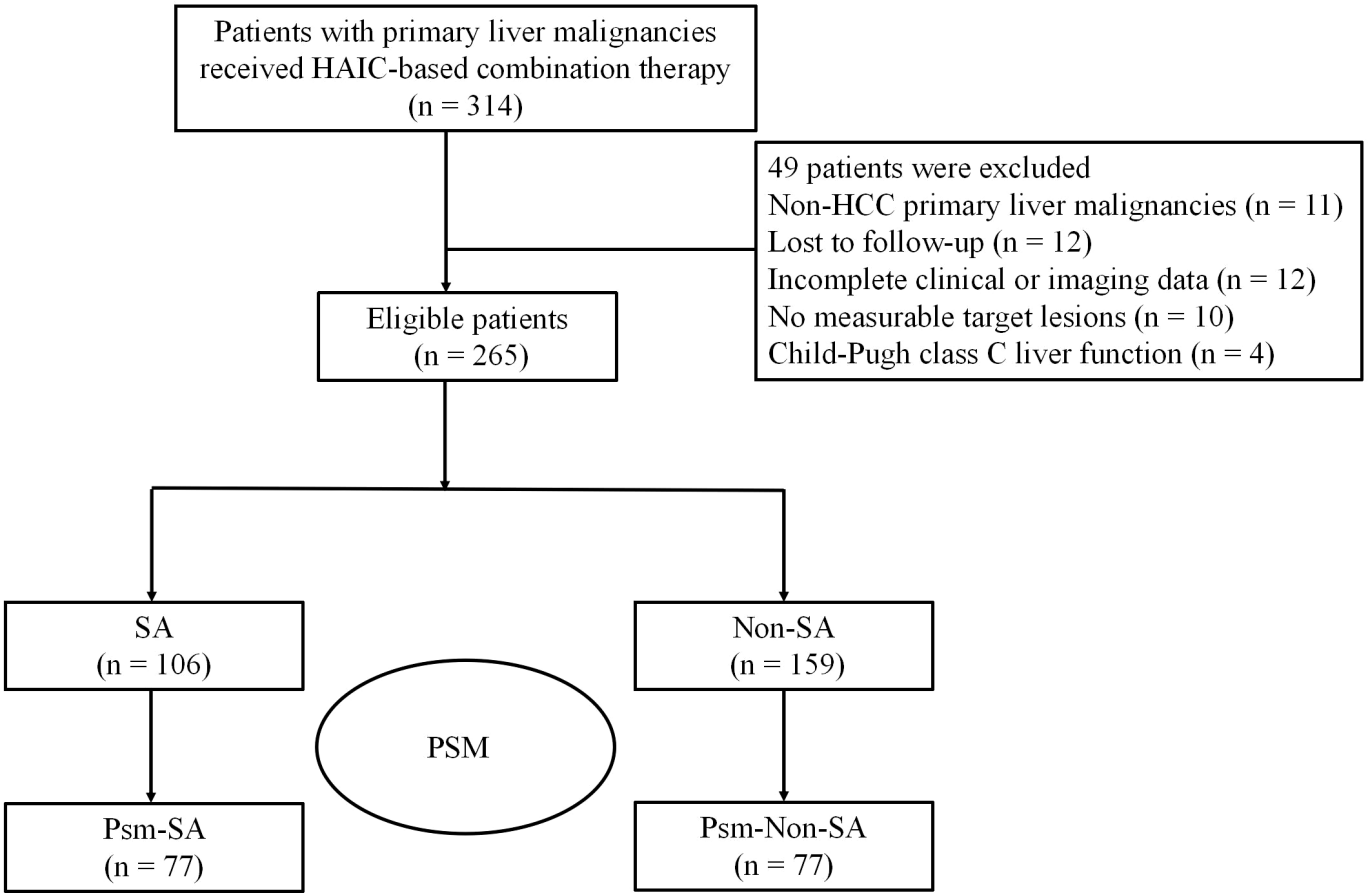
The flowchart of the study. SA, sarcopenia group; Non-SA, non-sarcopenia group; Psm-SA, propensity score-matched sarcopenia group; Psm-Non-SA, propensity score-matched non-sarcopenia group; HCC, hepatocellular carcinoma; HAIC, hepatic arterial infusion chemotherapy; PSM, propensity score matching.

This retrospective, single-center observational study included a total of 314 patients with unresectable HCC who received HAIC combined with targeted immunotherapy at Hepatic Surgery Center, Tongji Hospital, between August 2020 and December 2022. Inclusion criteria were: (1) pathologically confirmed HCC; (2) Eastern Cooperative Oncology Group (ECOG) performance status (PS) of 0-1; (3) Child-Pugh class A or B; and (4) assessed as surgically unresectable. Exclusion criteria included: non- HCC primary liver malignancies, loss to follow-up, incomplete clinical or imaging data, absence of measurable target lesions, and Child-Pugh class C. A final total of 265 patients met the eligibility criteria and were included in the analysis (**Figure 1**). All patients were followed up until January 24, 2024.

This study was approved by the Ethics Committee of Tongji Hospital, affiliated with Tongji Medical College, Huazhong University of Science and Technology (Approval No. TJ-IRB202403001). Informed consent was waived. This study was conducted in full accordance with the ethical standards of the Declaration of Helsinki and approved by the institutional review board.

### Treatment protocol

All patients were treated with HAIC using the mFOLFOX regimen (14), combined with programmed cell death protein-1 (PD-1) inhibitors and molecular targeted agents. The HAIC procedure was carried out in accordance with the “Expert Consensus on Arterial Infusion Chemotherapy for HCC in China” (15), with detailed steps outlined in **Supplementary Appendix 1**. HAIC was administered every 4–6 weeks and continued until disease progression, intolerable toxicity, or death. Immunotherapy andtargeted agents were given every 3 weeks, with at least three treatment cycles completed. Detailed drug information is provided in **Supplementary Appendix 2**.

### Handgrip strength assessment

Handgrip strength was measured using a digital dynamometer (model EH101, Xiangshan Camry Electronic Co., Ltd., Guangdong, China). Each participant was seated with the shoulder adducted and in a neutral rotation, the elbow flexed at 90°, and the forearm and wrist in a neutral position. Using their dominant hand, participants performed three maximum-effort grip trials with a one-minute interval between attempts. The highest value was recorded. According to the AWGS 2019 criteria, sarcopenia was defined as handgrip strength <28kg(male) and 18kg(female) (13).

### Efficacy and safety evaluation

All patients underwent contrast-enhanced CT or MRI every six weeks during treatment, along with hematological tests, to comprehensively evaluate therapeutic response and safety. Tumor response was evaluated by two independent radiologists based on the modified Response Evaluation Criteria in Solid Tumors (mRECIST) (16), categorized as complete response (CR), partial response (PR), stable disease (SD), or progressive disease (PD). Objective response rate (ORR) was calculated as the percentage of patients achieving CR or PR, while disease control rate (DCR) included patients with CR, PR, or SD. Overall survival (OS) was defined as the time from initiation of triple therapy to death or last follow-up, and progression-free survival (PFS) as the time from treatment initiation to either radiologic progression or death. The primary endpoint was PFS, while secondary endpoints included OS, ORR, and DCR. All treatment-related adverse events (AEs) were recorded and graded according to the National Cancer Institute Common Terminology Criteria for Adverse Events, version 5.0 (NCI-CTCAE v5.0) (17).

### Statistical analysis

All statistical analyses were performed using SPSS version 26.0 (IBM Corp., Armonk, NY, USA), and graphs were generated with GraphPad Prism version 9.5 (GraphPad Software, San Diego, CA, USA). Continuous variables were presented as mean ± standard deviation or median with interquartile range (IQR) and compared using the independent samples t-test or Mann-Whitney U test, as appropriate. Categorical variables were described as frequencies and percentages and compared using the chi- square test or Fisher’s exact test. Propensity score matching (PSM) was applied at a 1:1 ratio using a caliper width of 0.02 to balance baseline characteristics between groups. Kaplan-Meier curves were used to estimate OS and PFS, and differences were evaluated using the log-rank test. Univariate Cox proportional hazards models were performed to identify candidate predictors, and variables with *P* < 0.05 were further included in multivariate Cox regression to determine independent prognostic factors for OS and PFS. A two-sided *P* < 0.05 was considered statistically significant.

## Results

### Baseline characteristics of the patients

A total of 265 patients with unresectable HCC were included in this study. Baseline characteristics are summarized in **Table 1**. Prior to PSM, significant differences were observed between the sarcopenia group (SA group, n=106) and the non-sarcopenia group (Non-SA group, n=159) in several variables, including Eastern Cooperative Oncology Group performance status (ECOG PS), Child-Pugh class, prothrombin time, serum albumin, total bilirubin, tumor number, maximum tumor diameter, portal vein tumor thrombus, and extrahepatic metastasis (all *P* < 0.05). After PSM, the clinical characteristics were well balanced between the two groups (**Table 2**).

**Table 1:** Baseline characteristics of patients with hepatocellular carcinoma (before PSM).

Parameters	Total (n=265)(%)	SA (n=106)(%)	Non-SA (n=159)(%)	P
Gender				0.810
Male	236 (89. 1)	95 (89.6)	141 (88.7)	
Female	29 (10.9)	11 (10.4)	18 (11.3)	
Age, yr				0.909
≥ 60	69 (26.0)	28 (26.4)	41 (25.8)	
< 60	196 (74.0)	78 (73.6)	118 (74.2)	
ECOG PS				0.029
0	178 (67.2)	63 (59.4)	115 (72.3)	
≥ 1	87 (32.8)	43 (40.6)	44 (27.7)	
BCLC stage				0.198
A	25 (9.4)	7 (6.6)	18 (11.3)	
B-C	240 (90.6)	99 (93.4)	141 (88.7)	
Child-Pugh class				0.013
A	223 (84.2)	82 (77.4)	141 (88.7)	
B	42 (15.8)	24 (22.6)	18 (11.3)	
BMI, kg/m2				0.231
< 24	184 (69.4)	78 (73.6)	106 (66.7)	
≥ 24	81 (30.6)	28 (26.4)	53 (33.3)	
HBV-DNA, copies/ml				0.960
≥ 1000	122 (46.0)	49 (46.2)	73 (45.9)	
< 1000	143 (54.0)	57 (53.8)	86 (54. 1)	
Cirrhosis				0.330
Yes	231 (87.2)	95 (41. 1)	136 (58.9)	
No	34 (12.8)	11 (32.4)	23 (67.6)	
Previous treatment				0.497
Yes	71 (26.8)	26 (24.5)	45 (28.3)	
No	194 (73.2)	80 (75.5)	114 (71.7)	
PLT, *109/L				0.951
≤ 100	57 (21.5)	23 (21.7)	34 (21.4)	
> 100	208 (78.5)	83 (78.3)	125 (78.6)	
ALT, U/L				0.601
> 40	95 (35.8)	40 (37.7)	55 (34.6)	
≤ 40	170 (64.2)	66 (62.3)	104 (65.4)	
AST, U/L				0.394
> 40	177 (66.8)	74 (69.8)	103 (64.8)	
≤ 40	88 (33.2)	32 (30.2)	56 (35.2)	
ALB, g/L				0.004
< 35	79 (29.8)	42 (39.6)	37 (23.3)	
≥ 35	186 (70.2)	64 (60.4)	122 (76.7)	
TBIL, µmol/L				0.004
> 20	70 (26.4)	38 (35.8	32 (20. 1)	
≤ 20	195 (73.6)	68 (64.2)	127 (79.9)	
AFP, ng/ml				0.960
≥ 400	142 (53.6)	57 (53.8)	85 (53.5)	
< 400	123 (46.4)	49 (46.2)	74 (46.5)	
Tumor number				0.028
Multiple	156 (58.9)	71 (67.0)	85 (53.5)	
Single	109 (41. 1)	35 (33.0)	74 (46.5)	
Tumor diameter, cm				0.001
> 5	226 (85.3)	100 (94.3)	126 (79.2)	
≤ 5	39 (14.7)	6 (5.7)	33 (20.8)	
PVTT				0.019
Present	170(64.2)	77 (72.6)	93 (58.5)	
Absent	95 (35.8)	29 (27.4)	66 (41.5)	
EM				0.006
Present	53 (20.0)	30 (28.3)	23 (14.5)	
Absent	212(80.0)	76 (71.7)	136 (85.5)	

PSM, propensity score matching; SA, sarcopenia group; Non-SA, non-sarcopenia group; ECOG PS, Eastern Cooperative Oncology Group performance status; BCLC, Barcelona Clinic Liver Cancer; BMI, body mass index; HBV-DNA, hepatitis B virus deoxyribonucleic acid; PLT, platelet count; ALT, alanine aminotransferase; AST, aspartate aminotransferase; ALB, albumin; TBIL, total bilirubin; AFP, alpha-fetoprotein; PVTT, portal vein tumor thrombus; EM, extrahepatic metastasis.

Bold values means a P value < 0.05 was considered statistically significant.

**Table 2:** Baseline characteristic of patients with hepatocellular carcinoma (after PSM).

Parameters	Total (n=154)(%)	SA (n=77)(%)	Psm-Non-SA (n=77)(%)	P
Gender				0.632
Male	134 (87.0)	68 (88.3)	66 (85.7)	
Female	20 (13.0)	9 (11.7)	11(14.3)	
Age, yr				0.857
≥ 60	43 (27.9)	22 (28.6)	21 (27.3)	
< 60	111 (72. 1)	55 (71.4)	56 (72.7)	
ECOG PS				0.866
0	99 (64.3)	49 (63.6)	50 (64.9)	
≥ 1	55 (35.7)	28 (36.4)	27 (35. 1)	
BCLC stage				0.597
A	16 (10.4)	7 (9. 1)	9 (11.7)	
B-C	138 (89.6)	70 (90.9)	68 (88.3)	
Child-Pugh class				1.000
A	126 (81.8)	63 (81.8)	63 (81.8)	
B	28 (18.2)	14 (18.2)	14 (18.2)	
BMI, kg/m2				0.713
< 24	114 (74.0)	56 (72.7)	58 (75.3)	
≥ 24	40 (26.0)	21 (27.3)	19 (24.7)	
HBV-DNA, copies/ml				0.872
≥ 1000	77 (50.0)	39 (50.6)	38 (49.4)	
< 1000	77 (50.0)	38 (49.4)	39 (50.6)	
Cirrhosis				0.415
Yes	139 (90.3)	68 (88.3)	71 (92.2)	
No	15 (9.7)	9 (11.7)	6 (47.8)	
Previous treatment				0.857
Yes	43 (27.9)	21 (27.3)	22 (28.6)	
No	111 (72. 1)	56 (72.7)	55 (71.4)	
PLT, *109/L				0.536
≤ 100	29 (18.8)	13 (16.9)	16 (20.8)	
> 100	125 (81.2)	64 (83. 1)	61 (79.2)	
ALT, U/L				0.739
> 40	58 (37.7)	28 (36.4)	30 (39.0)	
≤ 40	96 (62.3)	49 (63.6)	47 (61.0)	
AST, U/L				1.000
> 40	108 (70. 1)	54 (70. 1)	54 (70. 1)	
≤ 40	46 (29.9)	23 (29.9)	23 (29.9)	
ALB, g/L				0.481
< 35	46 (29.9)	21 (27.3)	25 (32.5)	
≥ 35	108 (70. 1)	56 (72.7)	52 (67.5)	
TBIL, µmol/L				0.853
> 20	39 (25.3)	20 (26.0)	19 (24.7)	
≤ 20	115 (74.7)	57 (74.0)	58 (75.3)	
AFP, ng/ml				0.871
≥ 400	85 (55.2)	42 (54.5)	43 (55.8)	
< 400	69 (44.8)	35 (45.5)	34 (44.2)	
Tumor number				0.739
Multiple	96 (62.3)	49 (63.6)	47 (61.0)	
Single	58 (37.7)	28 (36.4)	30 (39.0)	
Tumor diameter, cm				0.415
> 5	139 (90.3)	71 (92.2)	68 (88.3)	
≤ 5	15 (9.7)	6 (7.8)	9 (11.7)	
PVTT				0.739
Present	96 (62.3)	49 (63.6)	47 (61.0)	
Absent	58 (37.7)	28 (36.4)	30 (39.0)	
EM				0.841
Present	31 (20. 1)	15 (19.5)	16 (20.8)	
Absent	123 (79.9)	62 (80.5)	61 (79.2)	

PSM, propensity score matching; SA, sarcopenia group; Non-SA, non-sarcopenia group; ECOG PS, Eastern Cooperative Oncology Group performance status; BCLC, Barcelona Clinic Liver Cancer; BMI, body mass index; HBV-DNA, hepatitis B virus deoxyribonucleic acid; PLT, platelet count; ALT, alanine aminotransferase; AST, aspartate aminotransferase; ALB, albumin; TBIL, total bilirubin; AFP, alpha-fetoprotein; PVTT, portal vein tumor thrombus; EM, extrahepatic metastasis.

Bold values means a P value < 0.05 was considered statistically significant.

### Tumor response

Before PSM, based on the mRECIST, the PR rate in the Non-SA group was 49. 1%, higher than 33.0% in the SA group (*P* = 0.010). ORR was significantly higher in the Non-SA group (53.5%) compared to the SA group (34.9%) (*P* = 0.003). DCR was 93.7% in the Non-SA group and 91.5% in the SA group, (*P* = 0.487). After PSM, the PR rate remained higher in the Non-SA group (49.4%) than in the SA group (35. 1%) (*P* = 0.073). The ORR in the Non-SA group was 54.5%, significantly greater than 37.7% in the SA group (*P* = 0.036). DCR was 94.2% and 96. 1% in the Non-SA and SA groups, respectively, with no statistically significant difference (*P* = 0.495) (**Table 3**).

**Table 3:** Tumor response in patients with hepatocellular carcinoma (Before and After PSM).

Before PSM				After PSM		
Response	SA (n=106)(%)	Non-SA (n=159)(%)	P	SA (n=77)(%)	Non-SA (n=77)(%)	P
CR	2 (1.9)	7 (4.4)	0.323	2 (2.6)	4 (5.2)	0.681
PR	35 (33.0)	78 (49. 1)	**0.010**	27 (35. 1)	38 (49.4)	0.073
SD	63 (59.4)	67 (42. 1)	**0.006**	45 (58.4)	2 (2.6)	**0.010**
PD	6 (5.7)	7 (4.4)	0.642	3 (3.9)	6 (7.8)	0.495
ORR	34.9%	53.5%	**0.003**	37.7%	54.5%	**0.036**
DCR	94.3%	95.6%	0.642	96. 1%	94.2%	0.495

PSM, propensity score matching; SA, sarcopenia group; Non-SA, non-sarcopenia group; Psm-SA, propensity score-matched sarcopenia group; Psm-Non-SA, propensity score-matched non-sarcopenia group; CR, complete response; PR, partial response; SD, stable disease; PD, progressive disease; ORR, objective response rate; DCR, disease control rate.

Bold values means a P value < 0.05 was considered statistically significant.

### Survival outcomes

**Figure 2:**
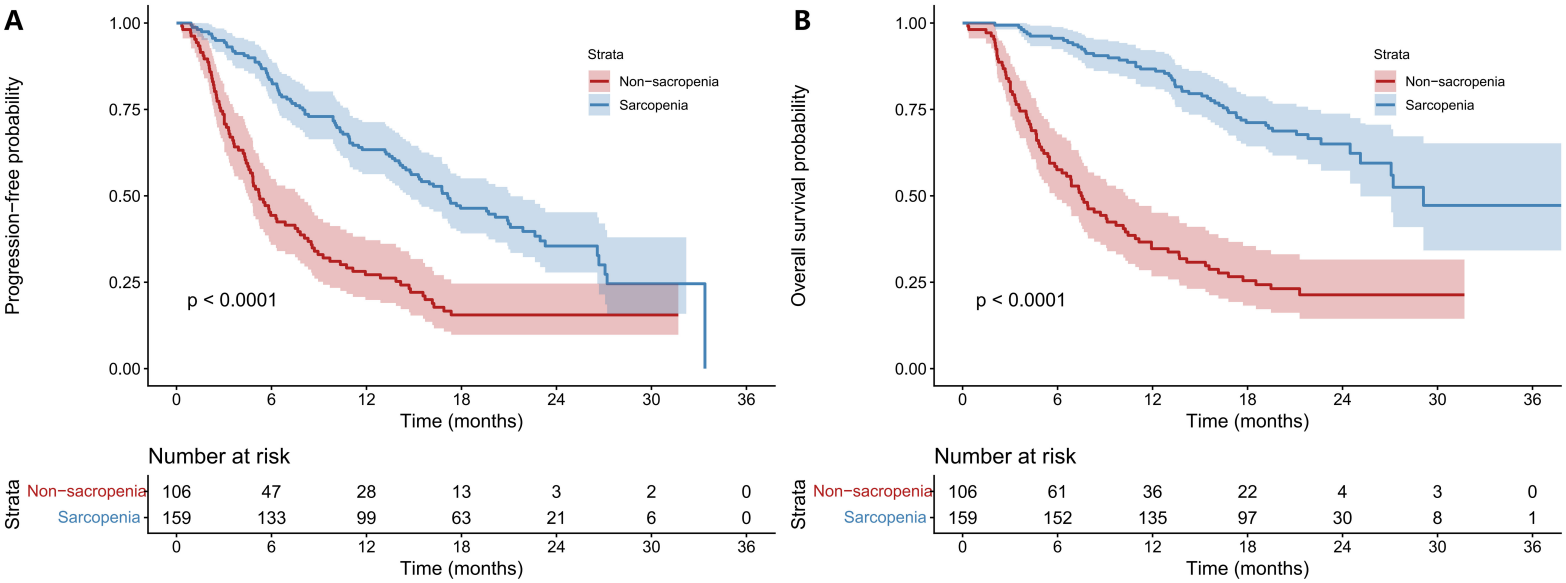
Comparisons of survival outcomes between sarcopenia and non-sarcopenia groups before PSM. Kaplan–Meier curves were plotted PFS **(A)** and OS **(B)** in patients with unresectable HCC receiving HAIC combined with immunotherapy and targeted therapy. The non-sarcopenia group exhibited superior survival outcomes. HCC: hepatocellular carcinoma; PFS: progression-free survival; OS: overall survival; HR: hazard ratio; CI: confidence interval; PSM: propensity score matching; HAIC: hepatic arterial infusion chemotherapy.

**Figure 3:**
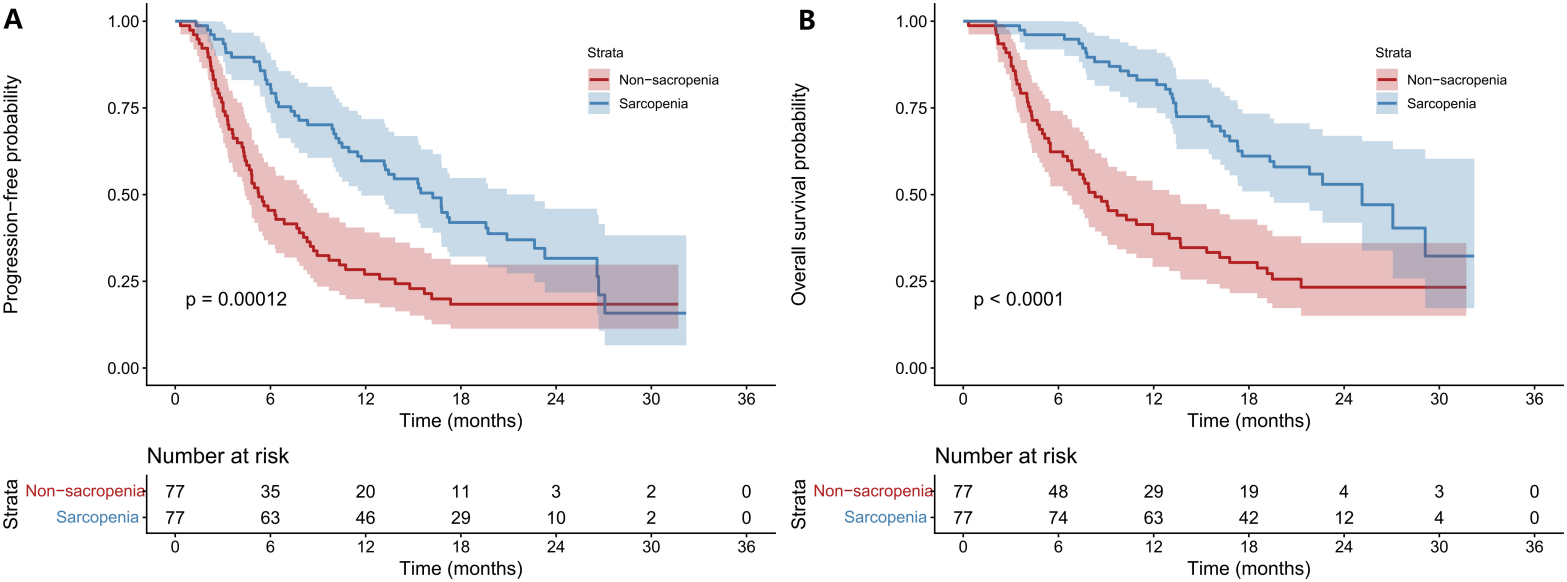
Comparisons of survival outcomes between sarcopenia and non-sarcopenia groups after PSM. Kaplan–Meier curves were plotted PFS **(A)** and OS **(B)** in patients with unresectable HCC receiving HAIC combined with immunotherapy and targeted therapy. The non-sarcopenia group consistently exhibited superior survival outcomes. HCC: hepatocellular carcinoma; PFS: progression-free survival; OS: overall survival; HR: hazard ratio; CI: confidence interval; PSM: propensity score matching; HAIC: hepatic arterial infusion chemotherapy.

Before PSM, mPFS was 17. 12 months in the Non-SA group, significantly longer than 5.22 months in the SA group (*P* < 0.001). mOS was 29.11 months in the Non-SA group versus 7.53 months in the SA group (*P* < 0.001)(**Figure 2**). After PSM, mPFS remained significantly longer in the Non-SA group (16.23 vs. 5.49 months, *P* < 0.001), as did mOS (25.13 vs. 8.31 months, *P* < 0.001), confirming that sarcopenia is associated with worse treatment outcomes (**Figure 3**). After PSM, the 6-, 12-, and 18- month PFS rates were 81.8%, 59.7%, and 37.7% in the Non-SA group, significantly higher than 46.8%, 27.3%, and 14.3% in the SA group (all *P* < 0.01). Similarly, the 6-, 12-, and 18-month OS rates were 96. 1%, 81.8%, and 55.8% in the Non-SA group, compared to 62.3%, 37.7%, and 23.4% in the SA group (all *P* < 0.001) (**Supplementary Appendix 3**).

### Risk factors analysis

**Table 4** shows that multivariate Cox regression analysis identified sarcopenia (HR: 2.30; 95% CI: 1.58–3.36; *P* < 0.001), high Hepatitis B virus DNA (HBV-DNA) levels (HR: 2.30; 95% CI: 1.53–3.46; *P* < 0.001), prior treatment (HR: 2. 15; 95% CI: 1.41–3.28; *P* < 0.001), and multiple tumors (HR: 2.27; 95% CI: 1.46–3.51; *P* < 0.001) as independent prognostic factors for PFS. For OS, significant factors included sarcopenia (HR: 3.76; 95% CI: 2.39–5.91; *P* < 0.001), ECOG PS ≥1 (HR: 1.73; *P* = 0.011), HBV-DNA ≥1000 copies/ml (HR: 1.62; *P* = 0.025), and multiple tumors (HR: 2.25; *P* = 0.001) (**Table 5**).

**Table 4:** Univariate and multivariate analyses of PFS.

Parameter	Univariate analysis	P	Multivariate analysis	P
	HR (95% CI)		HR(95% CI)	
ALB (< 35 vs. ≥ 35 g/L)	1.425(0.965~2. 104)	0.075		
TBIL (> 20 vs. ≤ 20 μmol/L)	1.039(0.678~ 1.592)	0.861		
AFP (≥ 400 vs. < 400 ng/ml)	0.977(0.675~ 1.412)	0.900		
Tumor number (Multiple vs. Single)	2.556(1.674~3.903)	**< 0.001**	2.265(1.464~3.507)	**< 0.001**
Tumor diameter (> 5 vs. ≤ 5 cm)	1.014(0.543~ 1.892)	0.966		
PVTT (Present vs. Absent)	1. 141(0.779~ 1.670)	0.498		
EM (Present vs. Absent)	1.494(0.963~2.317)	0.073		

PFS, progression-free survival; HR, hazard ratio ; CI, confidence interval ; ECOG PS, Eastern Cooperative Oncology Group performance status;BC, C, Barcelona Clinic, iver Cancer;BMI, body mass index ;HBV-DNA, hepatitis B virus deoxyribonucleic acid ;P, T, platelet ;A, T, alanine aminotransferase;AST, aspartate aminotransferase;A, B, albumin;TBI, , total bilirubin;AFP, alpha-fetoprotein;PVTT, portal vein tumor thrombus;EM, extrahepatic metastasis.

Bold values means a P value < 0.05 was considered statistically significant.

**Table 5:** Univariate and multivariate analyses of OS.

	Univariate analysis		Multivariate analysis	
Parameter	HR (95% CI)	P	HR (95% CI)	P
Gender (Male vs. Female)	0.918(0.527~ 1.599)	0.763		
Age (≥ 60 vs. < 60 yr)	0.778(0.485~ 1.248)	0.298		
Sarcopenia (Yes vs. No)	2.681(1.760~4.085)	**< 0.001**	3.757(2.388~5.912)	**< 0.001**
ECOG PS (≥ 1 vs. 0)	1.594(1.055~2.409)	**0.027**	1.729(1.134~2.635)	**0.011**
BCLC stage (B-C vs. A)	1.045 (0.551~ 1.983)	0.893		
Child-Pugh Class (B vs. A)	2. 104(1.308~3.385)	**0.002**	1.450(0.811~2.594)	0.210
BMI (< 24 vs. ≥ 24 kg/m2)	1.336(0.820~2. 177)	0.244		
HBV-DNA (≥ 1000 vs. < 1000 copies/ml)	1.802(1.190~2.727)	**0.005**	1.622(1.064~2.474)	**0.025**
Cirrhosis (Yes vs. No)	2. 120(0.925~4.857)	0.076		
Previous treatment (Yes vs. No)	1.383(0.897~2. 130)	0. 142		
PLT (≤ 100 vs. > 100 *109/L)	1.328(0.807~2. 185)	0.264		
ALT (> 40 vs. ≤ 40 U/L)	1.464 (0.971~2.207)	0.069		
AST (> 40 vs. ≤ 40 U/L)	1.572(0.987~2.505)	0.057		
ALB (< 35 vs. ≥ 35 g/L)	1.648(1.079~2.517)	**0.021**	1.630(0.974~2.727)	0.063
TBIL (> 20 vs. ≤ 20 μmol/L)	1.450(0.926~2.270)	0.104		
AFP (≥ 400 vs. < 400 ng/ml)	0.876(0.583~ 1.315)	0.523		
Tumor number (Multiple vs. Single)	2.235(1.406~3.552)	**0.001**	2.248(1.380~3.662)	**0.001**
Tumor diameter (> 5 vs. ≤ 5 cm)	0.982(0.509~ 1.893)	0.956		
PVTT (Present vs. Absent)	1.052(0.691~ 1.602)	0.813		
EM (Present vs. Absent)	1.745(1.101~2.767)	**0.018**	1.562(0.954~2.559)	0.076

PFS, progression-free survival; HR, hazard ratio ; CI, confidence interval ; ECOG PS, Eastern Cooperative Oncology Group performance status;BCLC, Barcelona Clinic Liver Cancer;BMI, body mass index ;HBV-DNA, hepatitis B virus deoxyribonucleic acid ;PLT, platelet ;ALT, alanine aminotransferase;AST, aspartate aminotransferase;ALB, albumin;TBIL, total bilirubin;AFP, alpha-fetoprotein;PVTT, portal vein tumor thrombus;EM, extrahepatic metastasis.

Bold values means a P value < 0.05 was considered statistically significant.

### Safety

AEs related to HAIC combined with targeted immunotherapy are summarized in **Supplementary Appendix 3**. Common grade 1–2 events included elevated aspartate aminotransferase (AST) levels (38.9%), thrombocytopenia (27.5%), anemia (27.2%), diarrhea (23.4%), and neutropenia (23.0%). Grade 3–4 toxicities were less frequent and primarily involved elevated AST (9.8%), thrombocytopenia (9.4%), neutropenia (7.5%), hyperbilirubinemia (5.7%), anemia (2.3%), and nausea/vomiting (1.5%).

## Discussion

This study of 265 patients with unresectable HCC receiving HAIC combined with targeted immunotherapy demonstrated significantly worse response rates and survival outcomes among those with sarcopenia, defined by low handgrip strength. ORR was lower in sarcopenic patients (37.7% vs. 54.5%), and mPFS and mOS were also significantly shorter (5.49 vs. 16.23 months; 8.31 vs. 25.13 months). Multivariate analysis confirmed sarcopenia as an independent predictor of both PFS and OS. These results highlight handgrip strength as a simple yet effective prognostic marker in the context of triple combination therapy.

While previous studies have linked sarcopenia to poor prognosis in HCC, they have largely relied on imaging-based measures like the skeletal muscle index (SMI) (18–20). Our findings demonstrate that handgrip strength, a more accessible clinical tool, offers similar prognostic value, especially for patients lacking imaging resources. Few studies have evaluated it in HAIC combined with targeted immunotherapy. Our study addresses this gap (21).

Sarcopenia may affect the efficacy of HAIC combined with immunotherapy through multiple mechanisms. First, as the primary reservoir of protein, muscle loss reflects poor nutritional status, which may compromise patients ‘ tolerance to chemotherapy and immunotherapy (22–24). Second, skeletal muscle is known to secrete various myokines that play essential roles in maintaining immune homeostasis and promoting T-cell activation (25, 26). A reduction in muscle mass may therefore impair antitumor immune responses and diminish the effectiveness of immune checkpoint inhibitors (23). Additionally, sarcopenic patients often exhibit chronic inflammatory states, such as elevated IL-6 and TNF-α levels, which may promote tumor progression and suppress immune response (27, 28). Collectively, these mechanisms suggest that decreased muscle strength not only reflects diminished nutritional and functional reserves but may also directly influence the tumor microenvironment and therapeutic responsiveness.

In our study, Previous treatment emerged as an independent predictor of worse PFS in patients receiving HAIC combined with targeted immunotherapy. This finding is clinically plausible for several reasons. First, patients who have received prior locoregional or systemic therapies often represent a subgroup with more advanced disease burden or biologically aggressive tumors, which intrinsically predisposes to poorer outcomes after subsequent lines of therapy (29, 30). Second, repeated locoregional interventions such as transarterial chemoembolization (TACE) can cause structural and functional changes to the hepatic arterial tree (including spasm, inflammation, and even occlusion) and may impair liver functional reserve, such changes can reduce the delivery efficiency and tolerability of subsequent HAIC (31, 32). Third, prior exposure to targeted agents or immune checkpoint inhibitors may select for resistant tumor subclones or promote immune-escape mechanisms (33, 34), thereby diminishing responsiveness to later targeted–immunotherapy combinations. Finally, a history of multiple previous treatments often correlates with cumulative liver injury and altered systemic and intratumoral immune landscapes, both of which may further compromise treatment efficacy (35). Taken together, these clinical and biological considerations provide a plausible explanation for the inferior PFS observed among previously treated patients and highlight the need to consider prior treatment history when stratifying patients and designing subsequent therapeutic strategies.

Nonetheless, this study has limitations. Its retrospective, single-center design introduces selection bias, and dynamic muscle strength data were unavailable. Sarcopenia was defined solely by grip strength without imaging-based confirmation, possibly underestimating occult cases. Inflammatory and immunologic markers were not analyzed. Future prospective multicenter studies should develop comprehensive risk models integrating functional, morphological, and metabolic indicators, and evaluate the role of muscle-strengthening interventions during immunotherapy.

## Conclusion

This study demonstrates that sarcopenia, defined by reduced handgrip strength, is independently associated with poorer treatment response, reduced survival, and higher recurrence in patients with unresectable HCC treated with HAIC combined with targeted immunotherapy.

## Data Availability

The original contributions presented in the study are included in the article/**Supplementary Material**. Further inquiries can be directed to the corresponding authors.
