# Supplementary material for: Prognostic significance of reduced handgrip strength in patients with unresectable hepatocellular carcinoma receiving HAIC combined with targeted immunotherapy
Source: Front Immunol. 2025 Dec 11;16:1672519. doi: 10.3389/fimmu.2025.1672519 (PMC12738834; doi:10.3389/fimmu.2025.1672519)
Supplement: Supplementary file 1 [file Table1.docx]

1. **Supplementary Appendix 1:**

**Interventional Procedure**
The patient was placed in the supine position. After anesthesia, the operator punctured the femoral artery using the Seldinger technique. To evaluate the tumor’s blood supply, digital subtraction angiography (DSA) was performed to visualize the anatomy of the celiac artery, superior mesenteric artery, and hepatic artery. Based on the vascular distribution and portal venous phase, an appropriate treatment plan was formulated. Depending on the tumor location, the catheter was inserted into either the left or right hepatic artery. A coaxial microcatheter was advanced superselectively into the tumor-feeding artery. In cases of large tumor volume or failure of superselective catheterization, the catheter was positioned in the right or left hepatic artery, with its tip placed to cover the entire arterial supply of the tumor as much as possible. After confirming optimal positioning via angiography, the catheter was secured in place. The distal end of the femoral sheath was coiled and externally fixed using medical adhesive film. A heparin cap and heparinized saline were applied to flush and seal the catheter to prevent thrombotic occlusion.

**Chemtherapy Infusion Protocol**After returning to the ward, patients received HAIC using the FOLFOX regimen. The FOLFOX protocol consisted of oxaliplatin at 85 mg/m² administered via continuous hepatic arterial infusion over 4 hours, leucovorin (calcium folinate) at 400 mg/m² administered via peripheral intravenous infusion over 3 hours, and 5-fluorouracil at 400 mg/m² continuously infused via the hepatic artery over 44 hours. Drug dosages were adjusted based on the patient's liver function status as assessed by the Child-Pugh classification and individual tolerance to chemotherapy. HAIC was typically repeated every 4 to 6 weeks and continued until disease progression, death, unacceptable toxicity, or a change in the treatment strategy.

**Supplementary Appendix 2:**

**Information on Immune Checkpoint Inhibitors (ICIs) and Molecular Targeted Agents**

| **Drug Category** | **Drug Name** | **Recommended Dose and Administration** |
| --- | --- | --- |
| ICIs |  |  |
| Camrelizumab | 200 mg, Jiangsu Hengrui | 200 mg per dose, every 3 weeks; intravenous infusion |
| Sintilimab | 100 mg, Innovent Biologics | 200 mg per dose, every 3 weeks; intravenous infusion |
| Tislelizumab | 100 mg, BeiGene | 200 mg per dose, every 3 weeks; intravenous infusion |
| Pembrolizumab | 100 mg, Merck & Co. | 200 mg per dose, every 3 weeks; intravenous infusion |
| Toripalimab | 240 mg, Junshi Biosciences | 240 mg per dose, every 3 weeks; intravenous infusion |
| Molecular Targeted Agents |  |  |
| Lenvatinib | 44 mg, Eisai, Japan | 8 mg once daily (body weight < 60 kg); 12 mg once daily (body weight ≥ 60 kg); oral administration |
| Donafenib | \| 0.1 g, Suzhou Zelgen \| \| --- \| | 0.2 g twice daily; oral administration |
| Apatinib | 425 mg, Jiangsu Hengrui | 850 mg once daily; oral administration |
| Sorafenib | 200 mg, Bayer, Germany | 0.4 g twice daily; oral administration |
| Regorafenib | 40 mg, Bayer, Germany | 80–160 mg once daily, orally for the first 21 days of each 28-day treatment cycle |

**Supplementary Appendix 3:**

**Table 4-6. Survival Outcomes of Patients with Intermediate to Advanced Hepatocellular Carcinoma Treated with HAIC Combined with Targeted Therapy and Immunotherapy**

| Before PSM | | | | |
| --- | --- | --- | --- | --- |
| Parameter | Patients（N=265） | SA（N=106） | Non-SA（N=159） | *P* value |
| 6m PFS（%） | 67.9 (180/265) | 44.3 (47/106) | 83.6 (133/159) | **<0.001** |
| 12mPFS（%） | 47.9 (127/265) | 26.4 (28/106) | 62.3 (99/159) | **<0.001** |
| 18mPFS（%） | 28.7 (76/265) | 12.3 (13/106) | 39.6 (63/159) | **<0.001** |
| Recurrence rate（%） | 70.2 (186/265) | 83.0 (88/106) | 61.6 (98/159) | **<0.001** |
| 6mOS （%） | 80.4 (213/265) | 57.5 (61/106) | 95.6 (152/159) | **<0.001** |
| 12mOS（%） | 64.5 (171/265) | 34.0 (36/106) | 84.9 (135/159) | **<0.001** |
| 18mOS（%） | 45.7 (121/265) | 20.8 (22/106) | 62.3 (99/159) | **<0.001** |
| Death rate（%） | 51.3 (136/265) | 76.4 (81/106) | 34.6 (55/159) | **<0.001** |
| Median follow up time（IQR，month） | 16.8 (7.6-21.9) | 19.6 (19.6-19.6) | 19.6 (17.9-20.4) | - |
| After PSM | | | | |
| Parameter | Patients（N=154） | Psm-SA（N=77） | Psm-Non-SA（N=77） | *P* value |
| 6m PFS（%） | 64.3 (99/154) | 46.8 (36/77) | 81.8 (63/77) | **<0.001** |
| 12mPFS（%） | 43.5 (67/154) | 27.3 (21/77) | 59.7 (46/77) | **<0.001** |
| 18mPFS（%） | 26.0 (40/154) | 14.3 (11/77) | 37.7 (29/77) | **0.001** |
| Recurrence rate（%） | 74.0 (114/154) | 80.5 (62/77) | 67.5 (52/77) | 0.066 |
| 6mOS （%） | 79.2 (122/154) | 62.3 (48/77) | 96.1 (74/77) | **<0.001** |
| 12mOS（%） | 59.7 (92/154) | 37.7 (29/77) | 81.8 (63/77) | **<0.001** |
| 18mOS（%） | 39.6 (61/154) | 23.4 (18/77) | 55.8 (43/77) | **<0.001** |
| Death rate（%） | 60.4 (93/154) | 74.0 (57/77) | 46.8 (36/77) | **0.001** |
| Median follow up time（IQR，month） | 15.4 (7.3-21.4) | 3.15 (3.15-3.15) | 13.2(8.3-13.2) | - |

**Supplementary Appendix 4**

**Table 4-9. Grading of Treatment-Related Adverse Events in Patients with Intermediate to Advanced Hepatocellular Carcinoma Treated with HAIC Combined with Targeted Therapy and Immunotherapy**

| Parameter | AEs grading | | |
| --- | --- | --- | --- |
|  | Totality（%） | Grade 1-2 (%) | Grade 3-4 (%) |
| Clinical symptoms | | | |
| Fatigue | 56 (21.1) | 56 (21.1) | 0 (0) |
| Pyrexia / Fever | 26 (9.8) | 26 (9.8) | 0 (0) |
| Nausea / Vomiting | 56 (21.1) | 52 (19.6) | 4 (1.5) |
| Hypertension | 3 (1.1) | 3 (1.1) | 0 (0) |
| Diarrhea | 62 (23.4) | 62 (23.4) | 0 (0) |
| Rash | 28 (10.5) | 28 (10.5) | 0 (0) |
| Proteinuria | 6 (2.26) | 6 (2.3) | 0 (0) |
| HRSR | 11 (4.2) | 11 (4.2) | 0 (0) |
| Anemia | 78 (29.4) | 72 (27.2) | 6 (2.3) |
| Laboratory examinations | | | |
| Neutropenia | 81 (30.5) | 61 (23.0) | 20 (7.5) |
| Thrombocytopenia | 98 (37.0) | 73 (27.5) | 25 (9.4) |
| Elevated Aspartate Aminotransferase (AST) | 129 (48.7) | 103 (38.9) | 26 (9.8) |
| Hyperbilirubinemia | 48 (18.1) | 33 (12.5) | 15 (5.7) |
| Hypoproteinemia | 75 (28.3) | 73 (27.5) | 2 (0.8) |
